# ﻿Two new species and one asexual morph record of *Paraisaria* (Ophiocordycipitaceae, Hypocreales) from China

**DOI:** 10.3897/mycokeys.121.156843

**Published:** 2025-09-01

**Authors:** Yu Yang, Kevin D. Hyde, Ausana Mapook, Yong-Zhong Lu, Somrudee Nilthong, Shu-Qiong Xie, Xiang-Dong Li, Ruvishika S. Jayawardena, Yuan-Pin Xiao

**Affiliations:** 1 School of Food and Pharmaceutical Engineering, Guizhou Institute of Technology, Guiyang 550025, China; 2 Guizhou Key Laboratory of Agricultural Microbiology, Guizhou Academy of Agricultural Sciences, Guiyang 550009, China; 3 Center of Excellence in Fungal Research, Mae Fah Luang University, Chiang Rai 57100, Thailand; 4 School of Science, Mae Fah Luang University, Chiang Rai, 57100, Thailand; 5 Department of Botany and Microbiology, College of Science, King Saud University, P.O. Box 22452, Riyadh 11495, Saudi Arabia; 6 Department of Plant Pathology, College of Agriculture, Guizhou University, Guiyang, Guizhou 550025, China; 7 Kyung Hee University, 26 Kyungheedae-ro, Dongdaemun-gu, Seoul, 02447, South Korea

**Keywords:** Entomopathogenic fungi, morphology, multi-locus, phylogeny, taxonomy

## Abstract

*Paraisaria* is a genus within Ophiocordycipitaceae, primarily parasitising insect groups such as ants (Hymenoptera), moth larvae (Lepidoptera) and beetle larvae (Coleoptera). The genus is characterised by cylindrical stipes, subglobose to globose fertile heads with immersed perithecia, hyaline, multi-septate ascospores and irregularly branched conidiophores with flask-shaped phialides and cylindrical to fusiform conidia. *Paraisaria* is globally distributed, primarily inhabiting tropical and subtropical locations; however, it has also demonstrated adaptability to temperate climates. This study introduces two novel species and reports one asexual morph of *Paraisaria* from China, providing detailed descriptions, illustrations and molecular phylogenetic analyses. Morphological examination reveals clear distinctions between the new species and previously described taxa. Multi-locus phylogenetic analyses (LSU, ITS, SSU, *tef*-1α, *rpb*1 and *rpb*2) corroborate their uniqueness, offering new insights into the diversity and evolutionary dynamics of the genus.

## ﻿Introduction

*Paraisaria*, a genus within Ophiocordycipitaceae (Hypocreales, Hypocreomycetidae, Sordariomycetes, Pezizomycotina, Ascomycota, Fungi, Hyde et al. (2024)), was established by Samson and Brady with *P. dubia* as the type species (Samson and Brady 1983). Its sexual morph was previously recognised as *Ophiocordyceps gracilis* (syn. *Cordyceps gracilis*) (Samson and Brady 1983). Early studies linked its asexual morphs to insect larvae and successfully isolated them from the sexual morphs of *Ophiocordyceps* (Samson and Brady 1983; Li et al. 2004; Sung et al. 2007). Initially, *Paraisaria* was proposed for suppression, favouring a broader concept of *Ophiocordyceps* under the “one fungus, one name” principle to unify sexual and asexual classifications (Quandt et al. 2014). However, molecular analyses revealed *Paraisaria* as a distinct monophyletic clade within the “*Ophiocordyceps ravenelii* subclade” (Sanjuan et al. 2015). *Paraisaria* was ultimately resurrected, segregated from *Ophiocordyceps* and amended to include sexual morphology, as proposed by Mongkolsamrit et al. (2019). Following this re-instatement, the genus has been further supported by the description of new species and combinations in subsequent studies (Wei et al. 2021; Tehan et al. 2023).

*Paraisaria* parasitises insect hosts, including cicada nymphs (Hemiptera), larvae of beetles (Coleoptera), flies (Diptera), moths (Lepidoptera) and ants (Hymenoptera), with habitats ranging from soil to leaf litter (Kobayasi 1941; Evans et al. 2010; Mongkolsamrit et al. 2019; Tehan et al. 2023). Geographically, *Paraisaria* has a broad distribution, predominantly in tropical and subtropical regions, such as Brazil, China and Argentina, but also occurs in temperate zones including parts of Europe and North America (Kobayasi 1941; Evans et al. 2010; Wen et al. 2016; Mongkolsamrit et al. 2019; Wei et al. 2021; Tehan et al. 2023). This wide range suggests adaptability to various ecological conditions, with a preference for warm and humid environments (Hennings 1904; Kobayasi 1941; Mongkolsamrit et al. 2019).

In addition to its ecological versatility, *Paraisaria* is defined by unique morphological features in both sexual and asexual forms. Sexual morphs are characterised by cylindrical, fleshy stipes, subglobose to globose fertile heads with immersed perithecia, cylindrical asci with thickened apical caps and hyaline, multi-septate ascospores fragmenting into cylindrical part-spores (Samson and Brady 1983; Mongkolsamrit et al. 2019; Tehan et al. 2023). Asexual morphs, documented in eight species, feature irregularly branched conidiophores with flask-shaped phialides and cylindrical, ellipsoid or fusiform conidia (Mongkolsamrit et al. 2019; Wei et al. 2021).

*Paraisaria* includes fungi that are important for ecology and the economy, yet their taxonomic diversity and ecological roles are still inadequately investigated (Mongkolsamrit et al. 2019; Tehan et al. 2023). Amongst the few known species, *Paraisaria gracilis* is particularly notable for its traditional use in Kazakh medicine and its anti-oxidative and antibacterial properties (Ma et al. 2012; Huang et al. 2019; Suo et al. 2014). Conversely, some species, such as *P. heteropoda*, pose public health risks due to their parasitism of edible insects, including cicadas (Doan et al. 2017). These fungi have been implicated in food poisoning outbreaks, including fatalities, caused by toxic mycotoxins, such as ibotenic acid (Doan et al. 2017; Tehan et al. 2023). Recent integrative analyses combining DNA sequencing with LC-HRMS techniques have further unveiled the genus’s chemical diversity (Tehan et al. 2023). Despite these advances, the taxonomic boundaries and species diversity within *Paraisaria* remain poorly resolved, hindering a comprehensive understanding of its ecological and economic potential (Doan et al. 2017; Mongkolsamrit et al. 2019; Tehan et al. 2023).

This study expands the understanding of *Paraisaria* by discovering two novel species and reports one asexual morph record from China. Morphological examinations revealed distinct traits that differentiate these species from known members of the genus. Phylogenetic analyses, based on six loci (LSU, ITS, SSU, *tef*-1α, *rpb*1 and *rpb*2), confirmed their novelty and classification within *Paraisaria*. These findings contribute to the growing knowledge of fungal diversity and highlight the evolutionary relationships within the genus, while also emphasising the need for further research on its ecological roles and life cycle mechanisms.

## ﻿Materials and methods

### ﻿Sample collection, macro- and micro- morphological examination

Six fresh specimens of *Paraisaria* species were collected from insect hosts in Anhui, Guizhou and Yunnan Provinces, China. Detailed metadata, including geographic coordinates and collection sites, were recorded during fieldwork (Rathnayaka et al. 2025). Those samples were then transported to the laboratory in plastic containers for further examination. In the laboratory, fruiting bodies were sectioned and examined using stereomicroscopes (Nikon SMZ 745 and SMZ 800N, Tokyo, Japan) to observe macroscopic features. Micromorphological traits, such as perithecia, asci, ascospores, synnemata, conidiophores, phialides and conidia, were documented using a Nikon DS-Ri2 digital camera attached to a Nikon ECLIPSE microscope, following the methodology outlined by Senanayake et al. (2020).

### ﻿Isolation and material deposition

A pure culture was obtained by transferring a small mass of mycelium from inside the host body to potato dextrose agar (PDA) with a flame-sterilised needle under aseptic conditions, then incubated at 25 °C in the dark. The resulting strains were deposited in the Guizhou Culture Collection (**GZCC**), China and dried specimens were deposited at the Herbarium of Cryptogams, Kunming Institute of Botany, Academia Sinica (**HKAS**). Morphological data were analysed using the Tarosoft (R) v.0.9.7 Image Framework and photographic images were produced and edited using Adobe Photoshop CC 2022 (Adobe Systems, USA). To ensure accurate taxonomic documentation, Facesoffungi and Index Fungorum numbers were assigned to the newly-described species, following the guidelines of Jayasiri et al. (2015) and https://www.indexfungorum.org/. The introduction of new species followed the protocols established by Maharachchikumbura et al. (2021) and Jayawardena et al. (2021).

### ﻿DNA extraction, PCR amplification and sequencing

Fungal genomic DNA was extracted from both dried samples and cultures using the E.Z.N.A.® Plant & Fungal DNA Kit (Omega Bio-Tek, USA) according to the manufacturer’s protocol. The extracted DNA was stored at -20 °C for future use. Four gene regions, internal transcribed spacers (**ITS**), large subunit rDNA (**LSU**), small subunit rDNA (**SSU**), transcription elongation factor 1-alpha gene region (***tef-1α***), largest subunit of RNA polymerase II (***rpb1***) and RNA polymerase II subunit (***rpb2***), were amplified and sequenced using primers listed in Table 1. PCR amplifications were performed in a 25 μl reaction volume containing 2 μl of DNA template, 8.5 μl of nuclease-free water, 1 μl of each primer (10 μM, final concentration 0.4 μM) and 12.5 μl of 2 × BenchTop™ Taq Master Mix (Biomiga, USA), which provides 1.25 units of Taq DNA polymerase per reaction. Primers were synthesised by Tsingke Biotech (Beijing, China). The PCR cycle included an initial denaturation at 98 °C for 2 minutes, followed by 40 cycles of 98 °C for 10 seconds, 55 °C for 1 minute and 72 °C for 30 seconds, with a final extension at 72 °C for 2 minutes. PCR products were examined by electrophoresis on a 1% (w/v) agarose gel in 1 × TAE buffer, stained with 4S Green Plus Nucleic Acid Stain (TSINGKE Biotech, China) and visualised under UV light. Agarose powder was purchased from Sangon Biotech (Shanghai, China) and sequences were obtained from Tsingke Biotechnology (Chongqing, China). Sequence assembly and editing were performed using BioEdit v.7.0.9 (Hall 1999). The resulting sequences were submitted to GenBank and their accession numbers are provided in Table 2.

**Table 1.** Sequences of primers used in this study.

Locus	Primers	Primer sequence (5′–3′)	References
ITS	ITS4	TCCTCCGCTTATTGATATGC	White et al. (1990)
	ITS5	GGAAGTAAAAGTCGTAACAAGG	
SSU	NS1	GTAGTCATATGCTTGTCTC	White et al. (1990)
	NS4	CTTCCGTCAATTCCTTTAAG	
LSU	LROR	ACCCGCTGAACTTAAGC	Vilgalys and Hester (1990)
	LR5	TCCTGAGGGAAACTTCG	
*tef*-1α	EF1-983F	GCYCCYGGHCAYCGTGAYTTYAT	Carbone and Kohn (1999); Rehner and Buckley (2005)
	EF1-2218R	ATGACACCRACRGCRACRGTYTG	
*rpb*1	CRPB1A	CAYCCWGGYTTYATCAAGAA	Castlebury et al. (2004)
	RPB1Cr	CCNGCDATNTCRTTRTCCATRTA	
*rpb*2	fRPB2-5f	GAYGAYMGWGATCAYTTYGG	Castlebury et al. (2004)
	fRPB2-7cR	CCCATRGCTTGYTTRCCCAT	

**Table 2.** Names, strain numbers, references and corresponding GenBank accession numbers of the taxa used in the phylogenetic analyses of this study.

Taxa names	Specimen/ Strain number	GenBank accession numbers						References
		LSU	ITS	SSU	*tef*-1α	*rpb*1	*rpb*2	
*Paraisaria alba*	HKAS 102484	MN943839	MN947219	MN943843	MN929085	MN929078	MN929082	Wei et al. (2021)
*Paraisaria amazonica*	HUA 186143	KJ917571	—	KJ917562	KM411989	KP212902	KM411982	Sanjuan et al. (2015)
*Paraisaria amazonica*	HUA 186113	KJ917572	—	KJ917566	—	KP212903	KM411980	Sanjuan et al. (2015)
*Paraisaria anhuiensis*	HKAS 132203	PV139238	PV139207	PV139224	PV156001	PV155972	PV155987	This study
*Paraisaria anhuiensis*	HKAS 132204	PV139239	PV139208	PV139225	PV156002	PV155973	PV155988	This study
*Paraisaria arcta*	HKAS 102553	MN943841	MN947221	MN943845	MN929087	MN929080	—	Wei et al. (2021)
*Paraisaria arcta*	HKAS 102552	MN943840	MN947220	MN943844	MN929086	MN929079	MN929083	Wei et al. (2021)
*Paraisaria blattarioides*	HUA186093	KJ917570	—	KJ917559	KM411992	KP212910	—	Sanjuan et al. (2015)
*Paraisaria blattarioides*	HUA 186108	KJ917569	—	KJ917558	—	KP212912	KM411984	Sanjuan et al. (2015)
*Paraisaria cascadensis*	OSC-M-052010	OQ708931	OQ709237	OQ800918	OR199814	OR199828	OR199838	Tehan et al. (2023)
*Paraisaria cascadensis*	OSC-M-052017	OQ708934	OQ709240	OQ800921	OR199817	OR199831	—	Tehan et al. (2023)
*Paraisaria coenomyiae*	NBRC 106964	AB968413	AB968397	AB968385	AB968571	—	AB968533	Ban et al. (2015)
*Paraisaria coenomyiae*	NBRC 108993	AB968412	AB968396	AB968384	AB968570	—	AB968532	Ban et al. (2015)
*Paraisaria coleopterorum*	HKAS 145895	PV139240	PV139209	—	PV156003	PV155974	PV155989	This study
*Paraisaria coleopterorum*	HKAS 145894	PV139241	PV139210	—	PV156004	PV155975	PV155990	This study
*Paraisaria dubia*	NJU985	—	—	MT918426	—	—	—	Yuan et al. (2022)
*Paraisaria gracilioides*	HUA186095	—	—	KJ917556	KM411994	KP212914	—	Sanjuan et al. (2015)
*Paraisaria gracilioides*	HUA 186092	KJ130992	—	KJ917555	—	KP212915	—	Sanjuan et al. (2015)
*Paraisaria gracilis*	EFCC 3101	EF468810	—	EF468955	EF468750	EF468858	EF468913	Sung et al. (2007)
*Paraisaria gracilis*	EFCC 8572	EF468811	JN049851	EF468956	EF468751	EF468859	EF468912	Sung et al. (2007)
*Paraisaria heteropoda*	OSC 106404	AY489722	—	AY489690	AY489617	AY489651	—	Quandt et al. (2014)
*Paraisaria heteropoda*	EFCC 10125	EF468812	JN049852	EF468957	EF468752	EF468860	EF468914	Sung et al. (2007)
*Paraisaria heteropoda*	NBRC 100643	JN941422	—	JN941719	AB968595	JN992453	AB968556	Ban et al. (2015)
*Paraisaria heteropoda*	BCC 18246	—	AB968411	AB113352	MK214083	MK214087	—	Mongkolsamrit et al. (2019)
*Paraisaria insignis*	OSC-M-052013	OQ708938	OQ709244	OQ800924	OR199820	OR199834	—	Tehan et al. (2023)
*Paraisaria insignis*	OSC-M-052004	OQ708927	OQ709234	OQ800914	OR199810	—	—	Tehan et al. (2023)
*Paraisaria monticola*	BPI 634610	—	OQ709246	—	—	—	—	Tehan et al. (2023)
*Paraisaria paramyrmicarum*	IMI 393961	EU797600	—	—	EU797597	—	—	Evans et al. (2010)
*Paraisaria orthopterorum*	BBC 88305	MK332583	MH754742	—	MK214080	MK214084	—	Mongkolsamrit et al. (2019)
*Paraisaria orthopterorum*	TBRC 9710	MK332582	MH754743	—	MK214081	MK214085	—	Mongkolsamrit et al. (2019)
*Paraisaria phuwiangensis*	TBRC 9709	MK192057	MK192015	—	MK214082	MK214086	—	Mongkolsamrit et al. (2019)
*Paraisaria phuwiangensis*	BBH 43491	MK192058	MH188542	—	—	MH211351	—	Mongkolsamrit et al. (2019)
*Paraisaria pseudoheteropoda*	OSC-M-052022	OQ708939	OQ709245	OQ800925	OR199821	OR199835	OR199841	Tehan et al. (2023)
*Paraisaria pseudoheteropoda*	OSC-M-052009	OQ708935	OQ709241	OQ800922	OR199818	OR199832	OR199840	Tehan et al. (2023)
*Paraisaria rosea*	HKAS_102546	MN943842	MN947222	MN943846	MN929088	MN929081	MN929084	Wei et al. (2021)
*Paraisaria* sp.	OSC-M-052011	OQ708932	OQ709238	OQ800919	OR199815	OR199829	OR199839	Tehan et al. (2023)
*Paraisaria* sp.	OSC-M-052026	OQ708936	OQ709242	—	—	—	—	Tehan et al. (2023)
*Paraisaria tettigonia*	GZUH CS14062709	—	—	KT345955	KT375440	KT375441	—	Wen et al. (2016)
*Paraisaria tettigoniae*	HKAS 144580	PV139242	PV139211	PV139226	PV156005	PV155976	PV155991	This study
*Paraisaria tettigoniae*	HKAS 132245	PV139244	PV139213	PV139228	PV156007	PV155978	PV155993	This study
*Paraisaria tettigoniae*	GZCC 24-0222	PV139243	PV139212	PV139227	PV156006	PV155977	PV155992	This study
*Paraisaria yodhathaii*	BBH 43163	MK332584	MH188539	—	MH211353	MH211349	—	Mongkolsamrit et al. (2019)
*Paraisaria yodhathaii*	TBRC 8502	MH201168	MH188540	—	MH211354	MH211350	—	Mongkolsamrit et al. (2019)
*Tolypocladium inflatum*	OSC 71235	EF469077	JN049844	EF469124	EF469061	EF469090	EF469108	Sung et al. (2007)
*Tolypocladium ophioglossoides*	NBRC 106332	JN941409	JN943322	JN941732	—	JN992466	MN929082	Schoch et al. (2012)

Note: The symbol “—” means that the sequence is not available and newly-generated sequences in this study are in bold.

### ﻿Phylogenetic analyses

The newly-generated sequences were assembled using SeqMan version 11.1.0 (DNASTAR, Inc., Madison, WI, USA), while reference and closely-related taxa for phylogenetic analysis were selected through BLAST searches on NCBI GenBank (https://blast.ncbi.nlm.nih.gov/Blast.cgi) and by reviewing relevant literature (Sung et al. 2007; Quandt et al. 2014; Ban et al. 2015; Sanjuan et al. 2015; Mongkolsamrit et al. 2019; Wei et al. 2021; Tehan et al. 2023) (Table 1). Phylogenetic inference included both previously published and newly-generated sequences. Sequence alignment for each nuclear locus region was conducted using the ‘auto’ option in MAFFT (Katoh and Standley 2013), followed by refinement using the ‘gappyout’ approach in TrimAl (Capella-Gutiérrez et al. 2009). The most appropriate nucleotide substitution models for each dataset were selected using the Bayesian Information Criterion (BIC), derived from a set of 22 commonly used DNA substitution models that incorporate rate heterogeneity, as implemented by ModelFinder (Kalyaanamoorthy et al. 2017). The aligned sequences were then concatenated and partitioning schemes were applied; further phylogenetic analysis was conducted.

Maximum Likelihood (ML) analyses were conducted using RAxML-HPC2 (Stamatakis 2014) on the CIPRES Science Gateway V. 3.3 (Miller and Blair 2009), with default settings, except for 1,000 bootstrap replicates. For Bayesian Inference (BI), the GTR+I+G nucleotide substitution model was selected as the best-fit model using MrModelTest 2.2 (Nylander 2004) and posterior probabilities (PP) were estimated using Markov Chain Monte Carlo (MCMC) sampling in MrBayes v.3.1.2 (Ronquist et al. 2012). The BI analysis was conducted using six simultaneous Markov chains, with trees sampled every 100 generations and ran for 5,000,000 generations, stopping once the average standard deviation of split frequencies dropped below 0.01. Convergence was verified using TRACER v.1.6 (Rambaut et al. 2013). The first 25% of the sampled trees were discarded as a burn-in period and the remaining trees were used to calculate PP. *Tolypocladium inflatum* (OSC 71235) and *T. ophioglossoides* (NBRC 106332) were chosen as outgroups. Significant support was determined as ML bootstrap values ≥ 75% and BI posterior probabilities ≥ 0.90. The final phylogenetic tree was visualised using FigTree v.1.4.0 (Rambaut 2012).

### ﻿Phylogenetic analysis results

**Figure 1.**
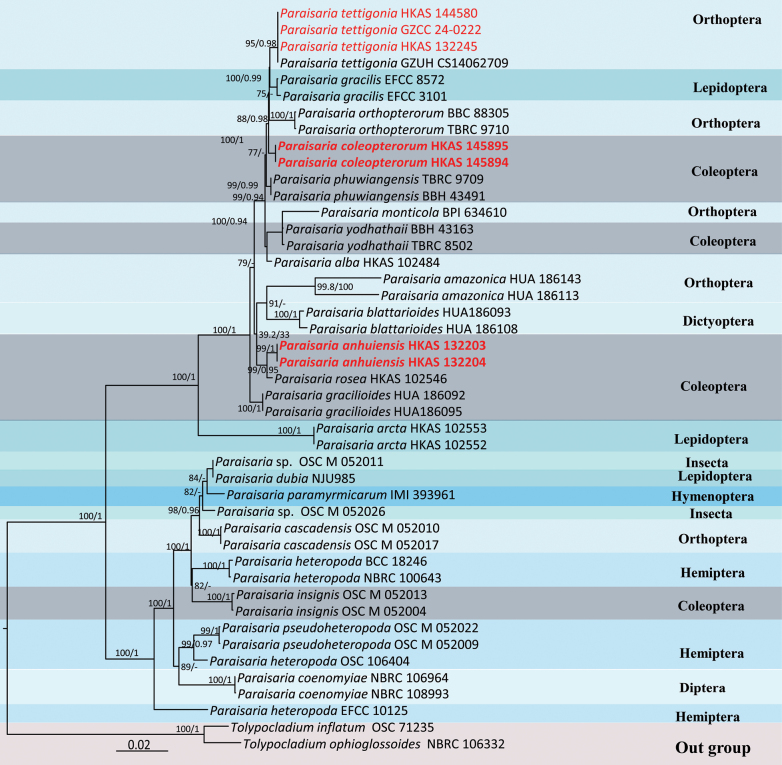
A phylogenetic tree was constructed using Maximum Likelihood (ML) analysis in RAxML, incorporating sequence data from multi-nuclear loci regions: LSU, ITS, SSU, *tef*-1α, *rpb*1 and *rpb*2. The analysis included *Tolypocladium inflatum* and *T. ophioglossoides* as outgroup taxa. Significant nodes, with ML bootstrap values equal to or greater than 75% and Bayesian posterior probabilities equal to or greater than 0.90, are indicated on the phylogram. Newly-generated sequences are emphasised in bold red for clarity.

The dataset combined LSU, ITS, SSU, *tef*-1α, *rpb*1 and *rpb*2 sequence data and encompassed 45 strains representing 26 taxa, with *Tolypocladium inflatum* (OSC 71235) and *T. ophioglossoides* (NBRC 106332) as outgroup taxa. It included 4845 aligned characters, distributed as follows: LSU (1–838 bp), ITS (839–1360 bp), SSU (1361–2343 bp), *tef*-1α (2344–3230 bp), *rpb*1 (3231–3885 bp) and *rpb*2 (3886–4845 bp). The tree topology of the RAxML analysis was consistent with that of the Bayesian analysis. The best-scoring RAxML tree had a final likelihood value of -16818.111708 (Fig. 1). The estimated base frequencies were A = 0.234476, C = 0.281939, G = 0.285069, T = 0.198516, with substitution rates as follows: AC = 1.237820, AG = 3.925347, AT = 0.890655, CG = 1.291471, CT = 7.097502, GT = 1.000000. The gamma distribution shape parameter α = 0.795567. The topologies from both the Maximum Likelihood (ML) and Bayesian analyses were manually reviewed and showed substantial agreement. Based on the phylogenetic results, two new species were recognised: *Paraisaria anhuiensis*, *P. coleopterorum* and one asexual morph record of *Paraisaria tettigoniae*.

## ﻿Taxonomy

### ﻿ Paraisaria anhuiensis

Taxon classificationFungiHypocrealesOphiocordycipitaceae

Y. P. Xiao, K.D. Hyde & Y. Yang sp. nov.

92BC5B35-6410-5152-B6AF-434DA11CD751

Index Fungorum: IF903775

Facesoffungi Number: FoF17627

Fig. 2

#### Etymology.

The epithet “*anhuiensis*” refers to the type location “Anhui Province, China”.

**Figure 2.**
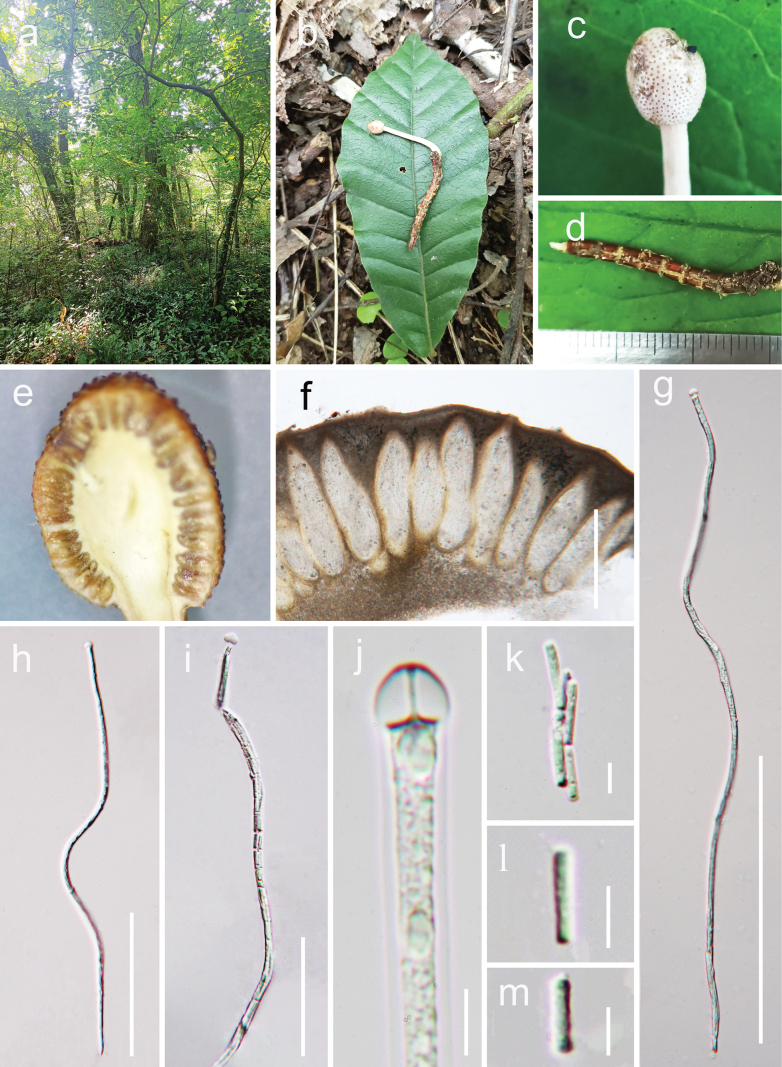
*Paraisaria anhuiensis* (HKAS 132203, holotype) a. Habitat; b. Overview of the host and stromata; c. Fertile head; d. Host; e, f. Vertical section of ascostroma; g–i. Asci; j. Apical cap; k–m. Secondary ascospores. Scale bars: 500 μm (f); 200 μm (g); 100 μm (h); 50 μm (i); 5 μm (j–m).

#### Holotype.

China • Anhui Province, Chuzhou City, occurs on the larvae of Coleoptera, on leaf litter, 191 m elev., 118.05°E, 32.33°N, 25 August 2021, Yu Yang, HFS29 (HKAS 132203, holotype).

#### Description.

***Parasitic*** on the larvae of Coleoptera. ***Host*** 2.1–3.3 long × 0.2–0.4 cm wide, reddish-brown, without hyphae on the surface. ***Sexual morph*: *Stromata*** 2.4–3.5 × 0.18–0.32 cm diam., mostly single, cylindrical, unbranched, emerging from the head of the larva body, yellowish-white. ***Fertile head*** 4.5 × 6 mm, subglobose, pale yellow when fresh, pale yellow-brown when dry, distinct from the stipe. ***Stipe*** 1.5–2.3 × 0.18–0.21 cm, pale yellow, straight, unbranched, glossy, cylindrical, inside not hollow. ***Perithecia*** 639–786 × 139–201 μm (*x̄* = 712.5 × 170 µm, n = 30), completely immersed, ampulliform, ostiolate, thick-walled. ***Asci*** 371–480 × 6.6–7.2 μm (*x̄* = 425.5 × 6.9 µm, n = 30), hyaline, filiform, with a thin apex. ***Apical cap*** 5.7–6.6 × 3.1–4.5 μm (*x̄* = 5.4 × 3.4 µm, n = 40), with a small channel in the centre. ***Ascospores*** filiform, equal to the asci in length, when mature, breaking into numerous secondary ascospores. ***Secondary ascospores*** 6.3–9.1 × 1.3–1.9 µm (*x̄* = 7.7 × 1.6, n = 40), cylindrical, one-celled, straight, hyaline, smooth. ***Asexual morph*** Not observed.

#### Other material examined.

China • Anhui Province, Huangshan City, parasitic on larvae of Coleoptera, on the soil, 403 m elev., 117.48E, 30.22N, 9 August 2023, Yu Yang, AH23190 (HKAS 132204, ***paratype***).

#### Notes.

The multi-locus phylogenetic analysis revealed that *Paraisaria anhuiensis* clusters with *P. rosea*, with 99% MLBP and 0.95 PP statistical support (Fig. 1). Morphologically, *Paraisaria anhuiensis* differs from *P. rosea* by producing longer asci (371–480 × 6.6–7.2 μm vs. 230–390 × 3.5–6 μm; L/W ratio 61.7 vs. 65.3) (Wei et al. 2021). *Paraisaria anhuiensis* differs from *P. rosea* in that its stromata emerge from the larval head and feature a pale-yellow fertile head, whereas *P. rosea* produces stromata from the middle part of the larval body, with a pink fertile head (Wei et al. 2021). Pairwise sequence comparison shows 2.16% (10/463 bp) in ITS, 0.58% (5/850) in *tef*-1α, 1.58% (10/632 bp) in *rpb*1 and 1.71% (17 out of 992 bp) in *rpb*2 between *P. anhuiensis* and *P. rosea* (Wei et al. 2021). Hence, we describe *Paraisaria anhuiensis* as a new species, based on its distinctive morphology and molecular evidence.

### ﻿ Paraisaria coleopterorum

Taxon classificationFungiHypocrealesOphiocordycipitaceae

Y. Yang, K.D. Hyde & Y. P. Xiao sp. nov.

40BD9C9D-3149-54D5-80E1-FB249CE98FAA

Index Fungorum: IF903776

Facesoffungi Number: FoF17628

Fig. 3

#### Etymology.

The epithet “*coleopterorum*” refers to its host belonging to the Coleoptera larvae.

**Figure 3.**
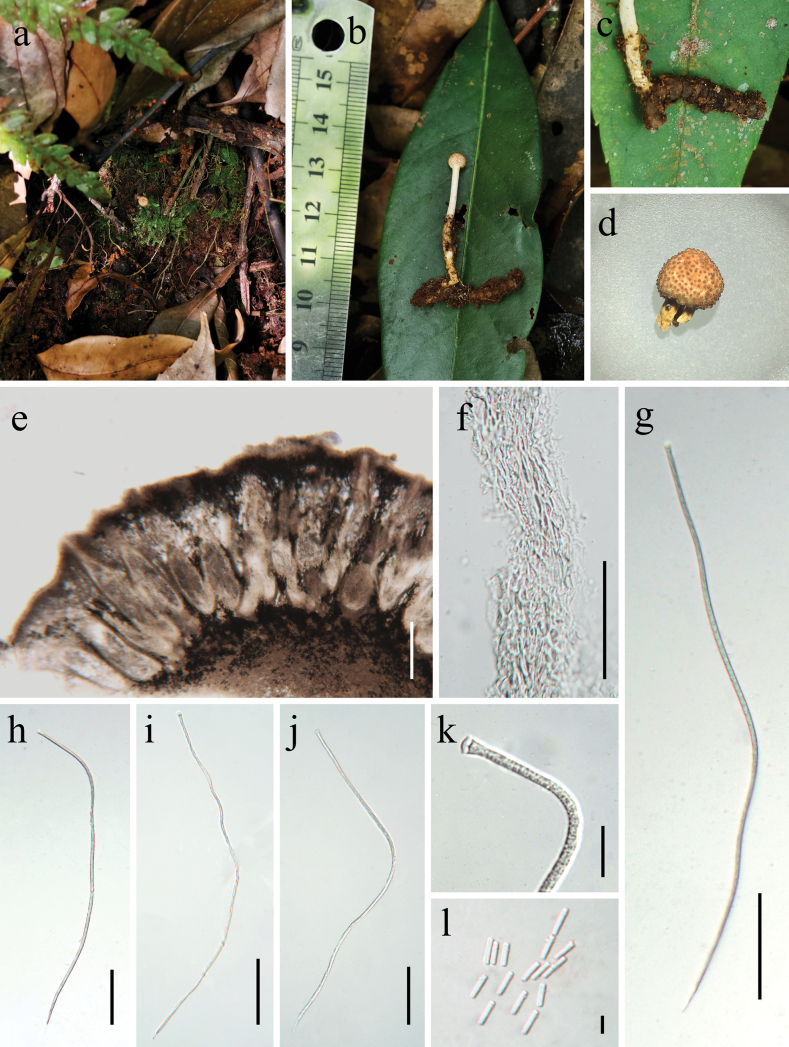
*Paraisaria coleopterorum* (HKAS 145895, holotype) a. Habitat; b. Overview of the host and stromata; c. Host; d. Fertile head; e. Vertical section of ascostroma; f. Peridium; g–j. Asci; k. Apical cap; l. Secondary ascospores. Scale bars: 200 μm (e); 50 μm (f); 100 μm (g–j); 20 μm (k); 5 μm (l).

#### Holotype.

China • Yunnan Province, Honghe Hani and Yi Autonomous Prefecture, Honghe County, parasitic on larva of Coleoptera, buried in the soil, 1963 m elev., 102.291E, 23.271N, 18 July 2024, Yu Yang, YY24340 (HKAS 145895, holotype)

#### Description.

***Parasitic*** on a larva of Coleoptera. ***Host*** 1.5–2.8 long × 0.3–0.5 cm wide, bark brown, without hyphae on the surface. ***Sexual morph Stromata*** 2.4–4.5 × 0.2–0.4 cm, typically solitary, cylindrical, unbranched, emerging from the larval body, simple, erect, pale yellowish-brown. ***Fertile head*** 4.5 × 5.6 mm, subglobose, pale yellowish-brown at the apex, becoming paler towards the base when fresh, turning pale pink when dry, distinctly separate from the stipe. ***Stipe*** 1.8–4 × 0.12–0.23 cm, pale yellow, straight, unbranched, glossy, cylindrical, with a solid interior. ***Perithecia*** 620–680 × 110–156 μm (*x̄* = 650 × 133 µm, n = 30), completely immersed, thick-walled. ***Peridium*** 22–36 (*x̄* = 29, n = 30) µm wide, comprising hyaline, three layers, ***textura porrecta*** outer layer forming a dense palisade layer covering the fertile head, ***textura intricata*** middle layer, ***textura porrecta*** inner layer. ***Asci*** 510–590 × 4.6–6.2 μm (*x̄* = 550 × 5.4 µm, n = 30), hyaline, cylindrical, with a thin apex. ***Apical cap*** 6.3–7.1 × 3.1–4.1 μm (*x̄* = 6.7 × 3.6 µm, n = 40). ***Ascospores*** equal in length to the asci, fragmenting into numerous secondary ascospores upon maturity. ***Secondary ascospores*** 6.4–8.1 × 1.6–2.3 µm (*x̄* = 7.2 × 1.9 µm, n = 40), cylindrical, one-celled, hyaline and smooth-walled. ***Asexual morph*** Not observed.

#### Other material examined.

China • Yunnan Province, Honghe Hani and Yi Autonomous Prefecture, Honghe County, parasitic on a larva of Coleoptera, buried in the soil, 1963 m elev., 102.291E, 23.271N, 18 July 2024, Yu Yang, YY24343 (HKAS 145894, ***paratype***).

#### Notes.

*Paraisaria coleopterorum* clustered with *P. gracilis*, *P. orthopterorum* and *P. phuwiangensis* in the phylogenetic tree with 88% MLBP, 0.98 PP support (Fig. 1). Pairwise sequence comparisons revealed differences of 1.47–2.75% (8–15/545) in ITS, 0.95–1.19% (8–10/836) in LSU, 0.76–1.30% (7–12/923) in *tef*-1α and 1.06–1.48% (10–14/943) in *rpb*1 between *P. coleopterorum* and *P. gracilis*/*P. orthopterorum*/*P. phuwiangensis*, respectively. The host of *P. coleopterorum* is the larva of Coleoptera, while *P. orthopterorum* infects Orthoptera nymphs (Mongkolsamrit et al. 2019). Compared to *P. orthopterorum*, *P. coleopterorum* produces longer and thinner perithecia (620–680 × 110–156 μm vs. 520–650 × 150–250 μm); L/W ratio 4.9 vs. 2.9) and longer asci (510–590 × 4.6–6.2 μm vs. 400 × 5 μm; L/W ratio 101.9 vs. 80) (Mongkolsamrit et al. 2019). When compared to *P. phuwiangensis*, *P. coleopterorum* has smaller perithecia (620–680 × 110–156 μm vs. 800–1200 × 300–380 μm; L/W ratio 4.9 vs. 2.9) and longer asci (510–590 × 4.6–6.2 μm vs. 500 × 3–5 μm; L/W ratio 101.9 vs. 125). Compared to *P. gracilis*, *P. coleopterorum* produces smaller perithecia (620–680 × 110–156 μm vs. 560–840 × 200–360 μm); L/W ratio 4.9 vs. 2.5) and longer asci (510–590 × 4.6–6.2 μm vs. 400–528 × 5–8 μm; L/W ratio 101.9 vs. 71.4) (Mongkolsamrit et al. 2019). Therefore, both morphological and phylogenetic analyses support the distinction of *P. coleopterorum* as a new species in *Paraisaria*.

### ﻿ Paraisaria tettigoniae

Taxon classificationFungiHypocrealesOphiocordycipitaceae

(T.C. Wen, Y.P. Xiao & K.D. Hyde) Luangsa-ard, Mongkols. & Samson [as ‘tettigonia’], Mycol. Progr. 18(9): 1225 (2019)

02E756DD-D752-59E8-8590-F2F1F33E2EDB

Index Fungorum: IF839725

Fig. 4

#### Description.

***Parasitic*** on adults of Orthoptera, found on the leaf litter. ***Host*** measuring 1.5–2.8 cm long, 5–8 mm wide, with hyphae present on the surface. ***Sexual morph Stromata*** 1.2–2.5 cm long, 2–5 mm wide, arising singly or in groups from the host prothorax stipitate, capitate, unbranched, yellowish-white to pale yellow when fresh, turning yellowish-brown when dry. ***Stipe*** 1–3.5 cm long, 1.2–1.8 mm diameter, yellowish white to pale yellow, cylindrical in shape, terminating in a fertile apex. ***Fertile head*** globoid, 1.5–4 mm, pale yellow and solitary. ***Perithecia*** 500–630 × 180–220 μm (*x̄*= 565 × 200 µm, n = 30), immersed, ovoid to flask-shaped, thick-walled. ***Asci*** 243–310 × 4.3–6.5 μm (*x̄*= 276 × 5.4 µm, n = 50), hyaline, cylindrical, with a thickened apex. ***Apical cap*** 4.9–6.7 × 3.4–4.4 μm (*x̄*= 5.8 × 3.9 µm, n = 50), thick, hyaline. ***Ascospores*** cylindrical, hyaline, as long as the asci, fragmenting into part-spores. ***Secondary ascospores*** 6.1–8.5 × 1.8–2.5 μm (*x̄*= 7.4 × 1.8 µm, n = 50) cylindrical, one-celled, straight, hyaline, smooth-walled. ***Asexual morph*** Hyphomycetous. ***Synnemata*** emerge from the insect body, white, 2–4 mm long, 0.2–0.5 mm wide. ***Conidiophores*** 21–43 μm long (*x̄* = 32 μm, n = 30), irregularly differentiated from the synnemata, sparse, gregarious and branched. ***Phialides*** 12–17 × 2.3–4.8 μm (*x̄*= 14.5 × 3.5 μm, n = 25), cylindrical with 1–3 necks, hyaline, aseptate, phialidic. ***Conidia*** 4.2–5.8 × 1.2–1.8 μm (*x̄* = 5 × 1.5 μm, n = 30), solitary, hyaline, aseptate, cylindrical with rounded tips, smooth-walled.

**Figure 4.**
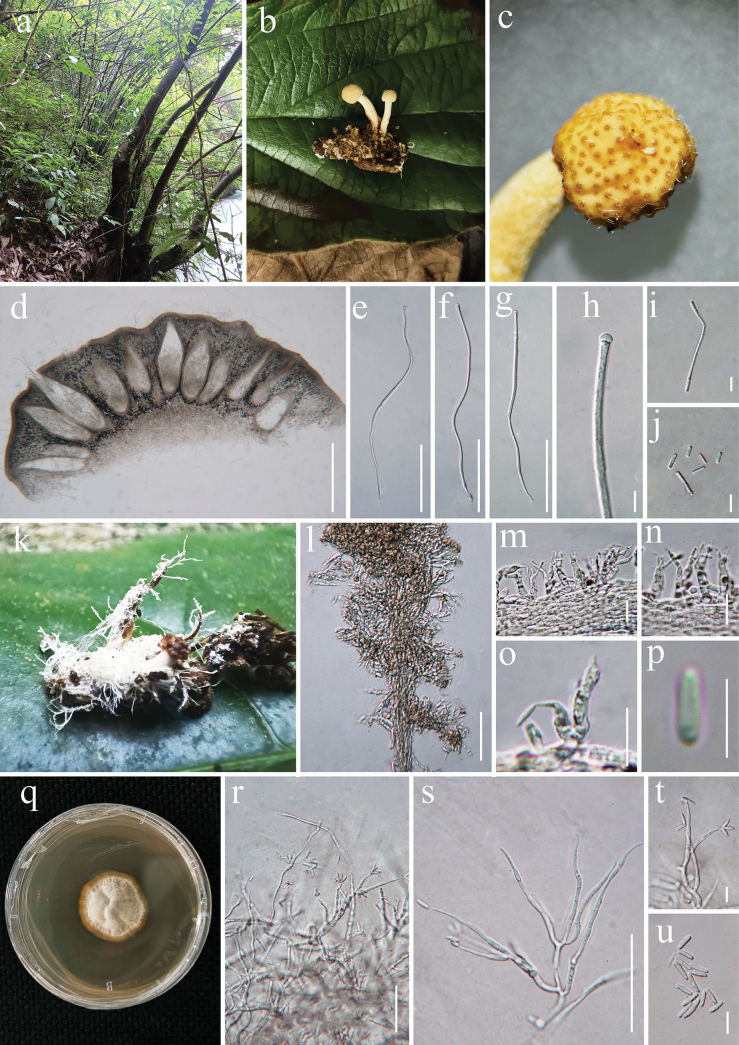
*Paraisaria tettigoniae* (HKAS 144580 and HKAS 132245). a. Habitat; b. Overview of the host and stromata; c. Stromata; d. Perithecia; e–g. Asci; h. Apical cap; i, j. Secondary ascospores; k. Synnemata on host; l. Synnemata; m, n. Phialides; p. Conidia; q. Culture; r, s. Conidiophores; t. Conidia on the phialides; u. Conidia. Scale bars: 500 μm (d); 100 μm (e–g); 50 μm (l, r–s); 10 μm (h–j, m–o, t–u); 5 μm (p).

#### Culture characteristics.

*Colonies* on PDA medium grow slowly, isolated from tissue taken inside the host body and are circular, reaching 2 cm in diameter after 35 days at 25 °C, with a white appearance. *Conidiophore* 50–90 μm (*x̄* = 70 µm, n = 40), bearing 2-4 phialides in one. *Phialides* 20–50 × 2.1–5.2 μm (*x̄* = 35 × 3.6 µm, n = 40) solitary, arising laterally from hyphae, hyaline, smooth. *Conidia* 7.8–14.5 × 2.1–3.2 μm (*x̄* = 11.1 × 2.6 µm, n = 40) hyaline, unicellular, ellipsoid, some slightly curved, smooth-walled.

#### Material examined.

China • Guizhou Province, Guiyang City, Xiuwen County, at 709 m elev., 27.256N, 106.674E, parasitic on adult of Orthoptera, collected on the leaf litter, 30 May 2024, Yu Yang XW2416 (HKAS 144580). XW2416J (GZCC 24-0222, living culture); China • Guizhou Province, Qiandongnan Miao and Dong Autonomous Prefecture, Zhen Yuan County, at 555 m elev., 27.111N, 108.401E, parasitic on an adult of Orthoptera, 26 June 2023, Yu Yang, TX23108 (HKAS 132245).

#### Notes.

Our new collection is phylogenetically closely related to *Paraisaria tettigoniae*, with 95% MLBP and 0.98 PP support (Fig. 1). It shares highly similar sequences with *P. tettigoniae* across multiple loci (SSU, *tef*-1α and *rpb*1), whereas *P. tettigoniae* shows anomalous divergence. Further inspection indicates that the ITS sequence of *P. tettigoniae* (GenBank accession: KT345954) may be problematic, possibly due to sequencing errors or intragenomic variation. Therefore, this ITS sequence (strain GZUH CS14062709) was excluded from our analysis. *Paraisaria tettigoniae* was originally described from Guizhou, China, based on its sexual morph parasitising adult Orthoptera (Wen et al. 2016). Our collection, also from Guizhou, represents the asexual morph of the same species. Although minor morphological differences were observed — such as smaller perithecia (500–630 × 180–220 μm vs. 520–680 × 205–275 μm) and shorter asci (243–310 × 4.3–6.5 μm vs. 530–615 × 6.5–9.3 μm) — the multi-locus phylogenetic analysis (excluding the problematic ITS sequence) shows that our collection clusters with *P. tettigoniae* (Fig. 1). Therefore, based on both morphological characteristics and multi-locus phylogenetic evidence, our collection is identified as *Paraisaria tettigoniae*, representing the first report of its asexual morph.

## ﻿Discussion

Taxonomic studies on *Paraisaria* in China remain underexplored compared to other fungal groups (Yang et al. 2021, 2024; Xiao et al. 2023, 2024). Until the present study, only three *Paraisaria* new species have been described from China, based on morphological and molecular evidence (Wen et al. 2016; Wei et al. 2021). Wen et al. (2016) introduced *Ophiocordyceps tettigonia* from Guizhou Province, China, which was subsequently transferred to *Paraisaria*, based on multi-gene phylogenetic analysis and morphological characterisation (Wen et al. 2016; Mongkolsamrit et al. 2019). Subsequently, Wei et al. (2021) described *Paraisaria arcta* from Guizhou Province and *P. rosea* from Yunnan Province. These findings highlight the limited exploration of the taxonomic diversity of the genus in China. We describe two new species of *Paraisaria* (*P. anhuiensis* and *P. coleopterorum*) and report one asexual morph of *Paraisaria tettigoniae* using an integrative approach that combines morphological characteristics with phylogenetic analyses. These newly-recognised taxa, including two new species and one asexual morph record, are each placed in well-supported clades within the phylogenetic tree (Fig. 1).

*Paraisaria* fungi exhibit remarkable parasitic versatility, infecting a diverse range of insect hosts across multiple orders, including Coleoptera, Dictyoptera, Diptera, Lepidoptera, Orthoptera, Hemiptera and Hymenoptera (Kobayasi 1941; Evans et al. 2010; Mongkolsamrit et al. 2019; Wei et al. 2021; Tehan et al. 2023). Most species in this genus predominantly parasitise Orthoptera and Coleoptera, as reflected in the phylogenetic tree (Fig. 1). *P. anhuiensis* and *P. coleopterorum* form well-supported, separate clades in the phylogenetic tree (highlighted in red; Fig. 1), demonstrating their molecular distinctiveness. These placements, together with their diagnostic morphological differences, provide robust support for their recognition as independent species. Furthermore, detailed morphological comparisons reveal significant diagnostic differences, which reinforce their unique taxonomic identities and support their classification as distinct species. In addition, the asexual morph of *P. tettigoniae* is reported here for the first time, enriching our understanding of its life cycle and expanding the known morphological diversity within *Paraisaria*.

The discovery of two new species and the report of one asexual morph record in this study significantly expands our understanding of the taxonomic diversity within *Paraisaria*. Previous studies have highlighted the ecological and economic importance of the genus, including its anti-oxidative and antibacterial properties (Ma et al. 2012; Suo et al. 2014; Huang et al. 2019), as well as its potential risks to public health due to mycotoxin production (Doan et al. 2017; Tehan et al. 2023). However, the taxonomic boundaries and species diversity of *Paraisaria* have remained poorly resolved, hindering further exploration of its potential applications. Our findings provide a foundation for future studies to investigate the ecological roles, chemical diversity and functional genomics of these newly-described species. Future studies could integrate multi-omics approaches, such as metabolomics and transcriptomics, to further explore the ecological roles and functional potential of *Paraisaria*.

## Supplementary Material

XML Treatment for Paraisaria anhuiensis

XML Treatment for Paraisaria coleopterorum

XML Treatment for Paraisaria tettigoniae

## ﻿Additional information

### Use of AI

No use of AI was reported.

### Author ORCIDs

Yu Yang https://orcid.org/0000-0001-8268-487X

Kevin D. Hyde https://orcid.org/0000-0002-2191-0762

Ausana Mapook https://orcid.org/0000-0001-7929-2429

Yong-Zhong Lu https://orcid.org/0000-0002-1033-5782

Somrudee Nilthong https://orcid.org/0000-0002-7454-5826

Shu-Qiong Xie https://orcid.org/0009-0006-8011-7680

Xiang-Dong Li https://orcid.org/0009-0001-4404-1803

Ruvishika S. Jayawardena https://orcid.org/0000-0001-7702-4885

Yuan-Pin Xiao https://orcid.org/0000-0003-1730-3545

### Data availability

All of the data that support the findings of this study are available in the main text or Supplementary Information.

## Supplementary materials

Supplementary material 1 Supplementary images Data type docx https://binary.pensoft.net/file/1406363This dataset is made available under the Open Database License (http://opendatacommons.org/licenses/odbl/1.0/). The Open Database License (ODbL) is a license agreement intended to allow users to freely share, modify, and use this Dataset while maintaining this same freedom for others, provided that the original source and author(s) are credited.Yu Yang, Kevin D. Hyde, Ausana Mapook, Yong-Zhong Lu, Somrudee Nilthong, Shu-Qiong Xie, Xiang-Dong Li, Ruvishika S. Jayawardena, Yuan-Pin Xiao
