# Supplementary material for: ﻿Two new species and one asexual morph record of Paraisaria (Ophiocordycipitaceae, Hypocreales) from China
Source: MycoKeys. 2025 Sep 1;121:253–70. doi: 10.3897/mycokeys.121.156843 (PMC12418028; doi:10.3897/mycokeys.121.156843)
Supplement: Supplementary material 1 — Supplementary images [file mycokeys-121-253-s001.docx]

Supplementary Figures S1–S5


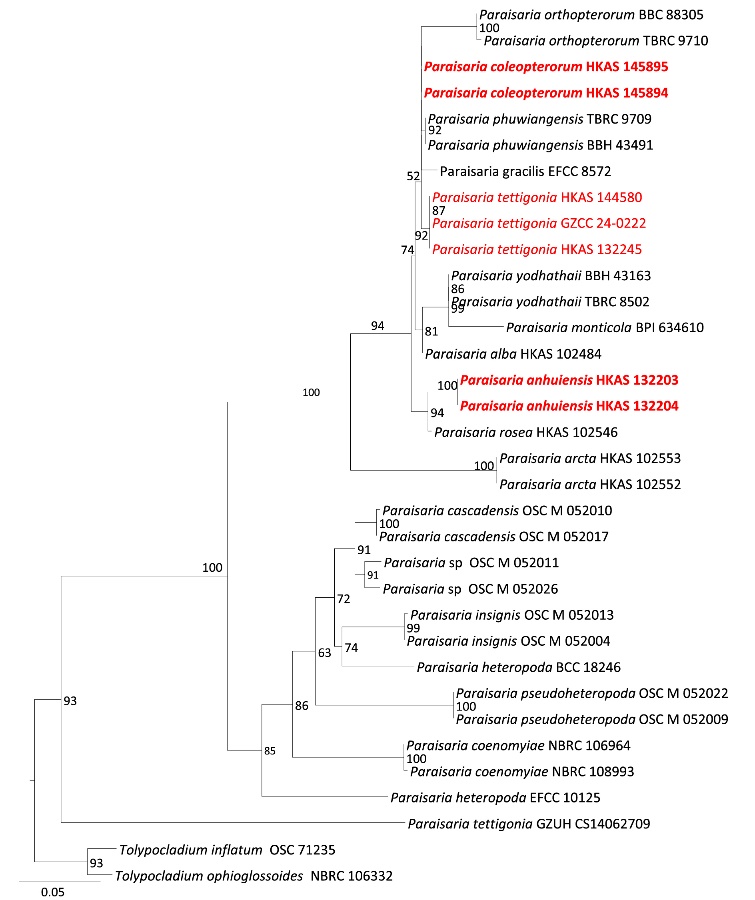


S1. A phylogenetic tree was constructed using maximum likelihood (ML) analysis in RAxML, incorporating sequence data from ITS


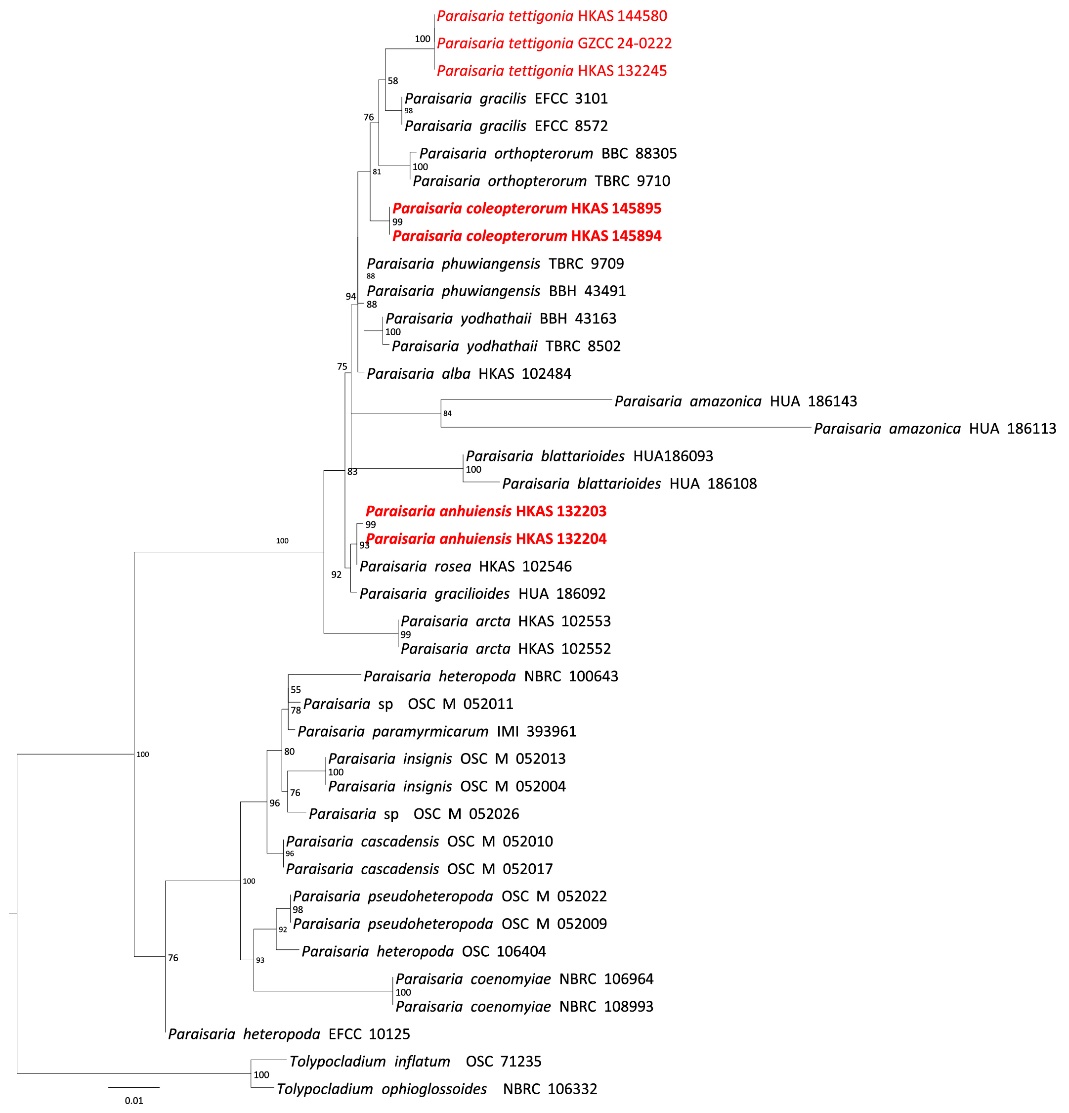


S2. A phylogenetic tree was constructed using maximum likelihood (ML) analysis in RAxML, incorporating sequence data from LSU


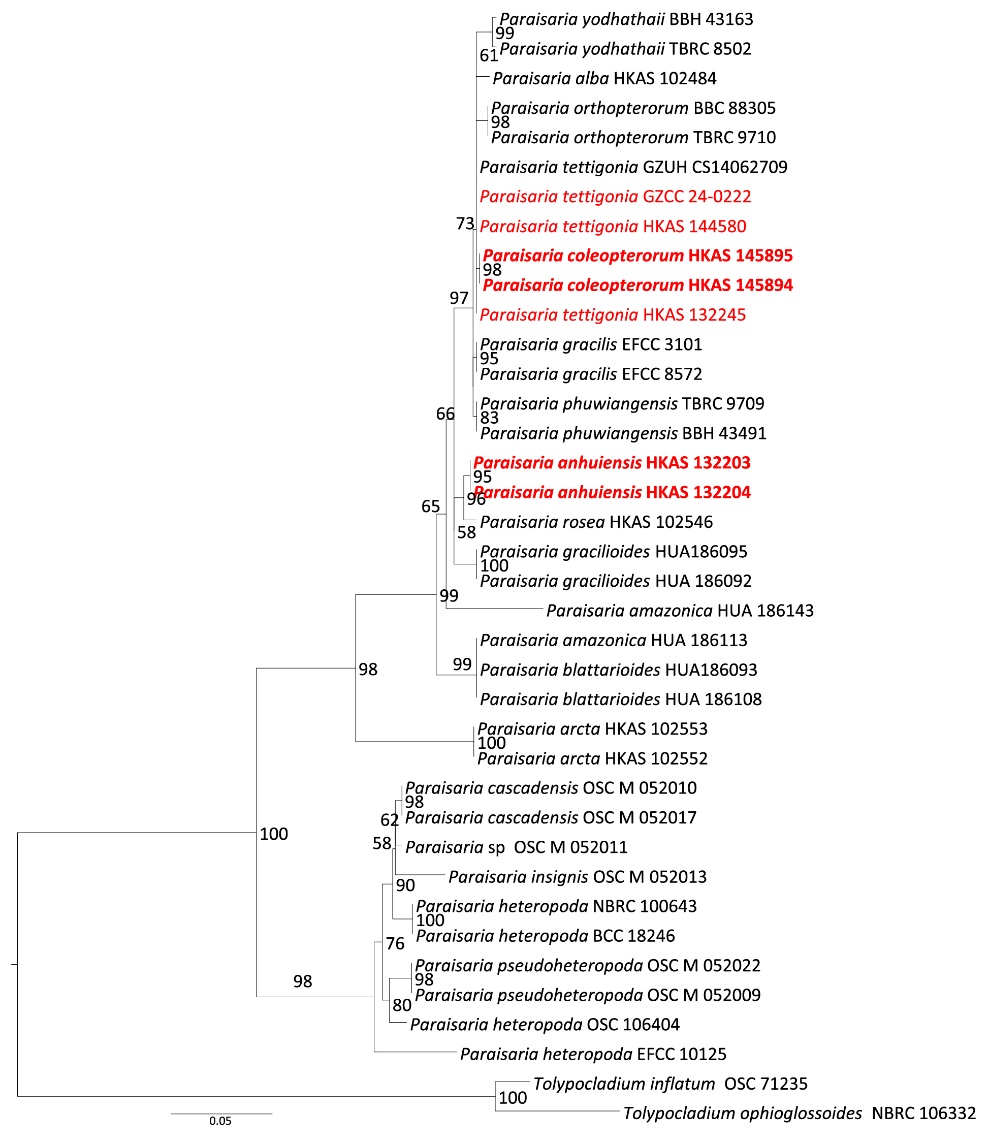


S3. A phylogenetic tree was constructed using maximum likelihood (ML) analysis in RAxML, incorporating sequence data from RPB1


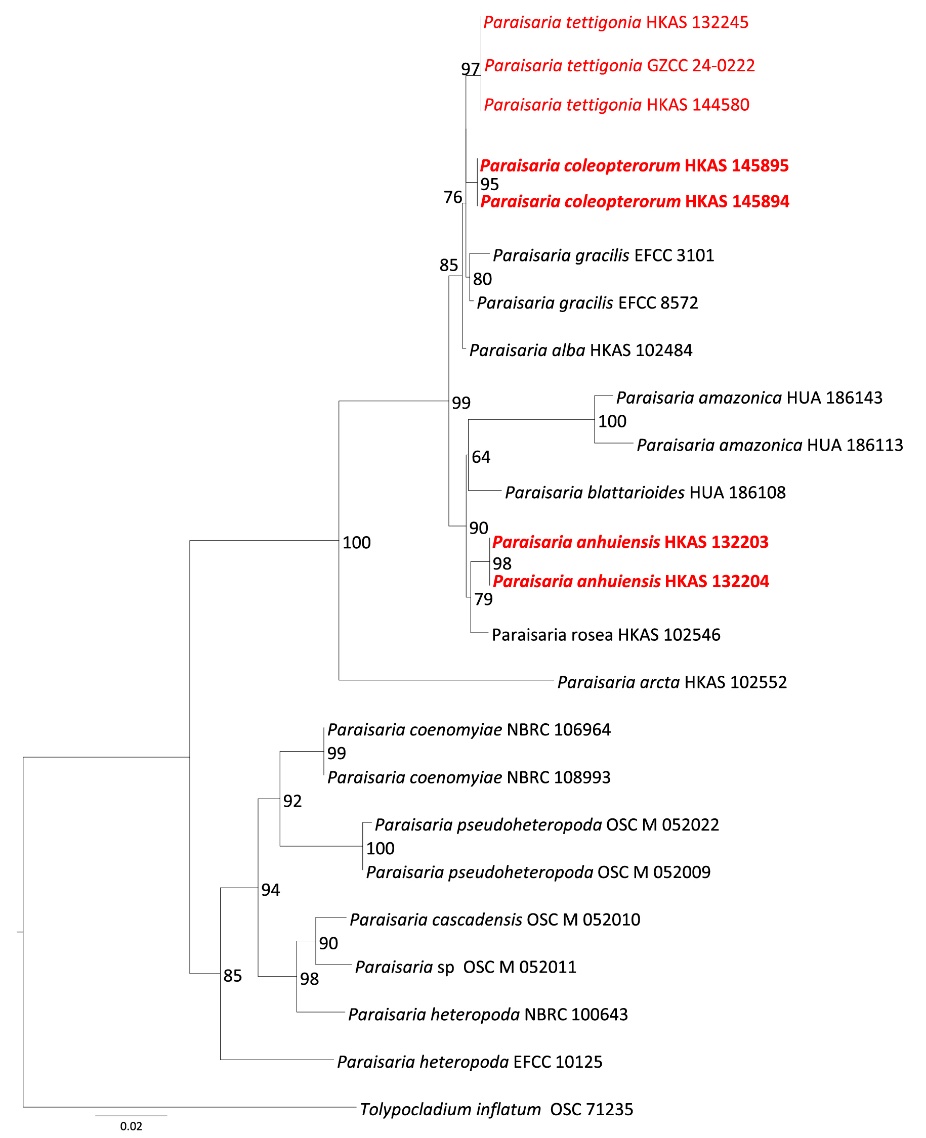


S4. A phylogenetic tree was constructed using maximum likelihood (ML) analysis in RAxML, incorporating sequence data from RPB2


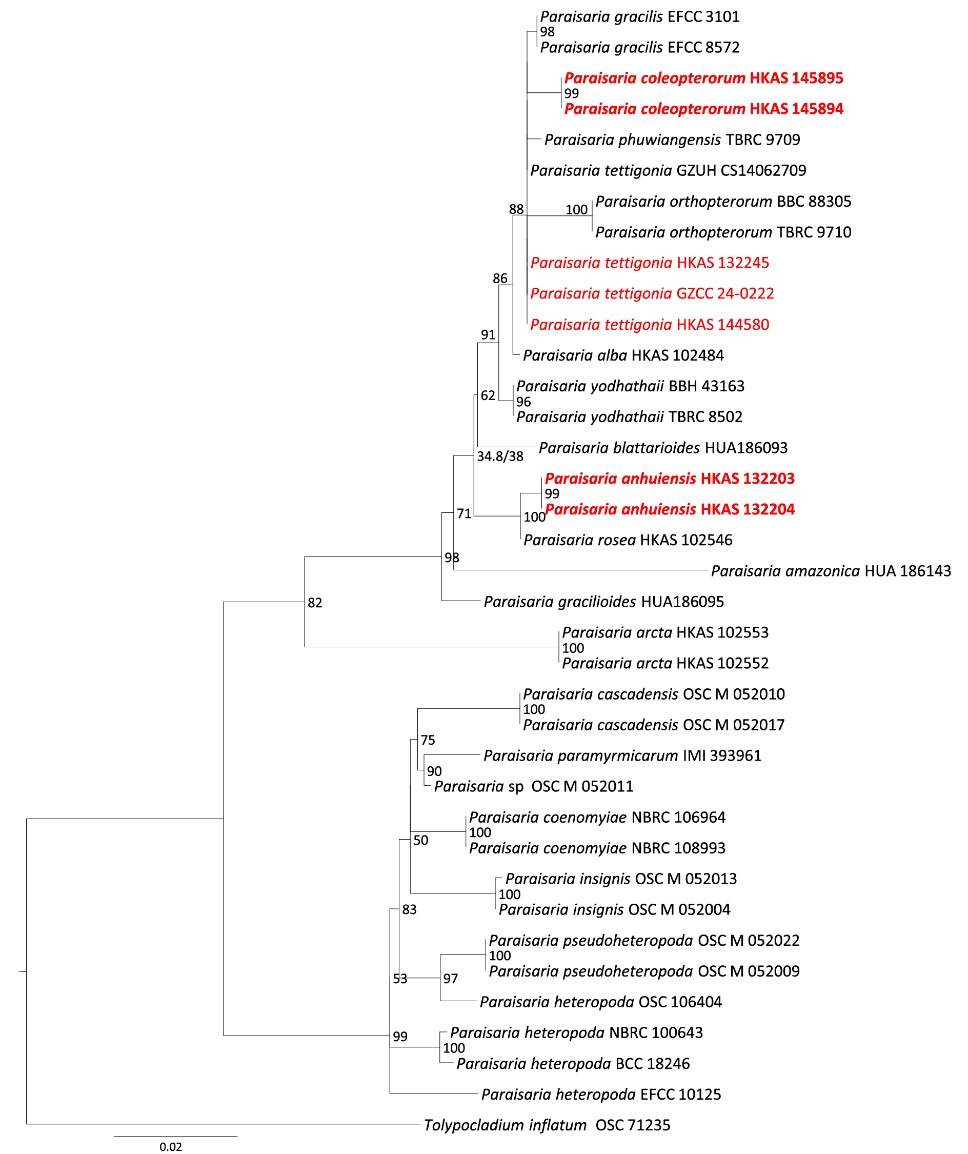


S5. A phylogenetic tree was constructed using maximum likelihood (ML) analysis in RAxML, incorporating sequence data from TEF1-α
